# 4,4′-Bipyridinium 1,4-phenyl­ene­diacetate

**DOI:** 10.1107/S1600536809036836

**Published:** 2009-09-19

**Authors:** Maomao Jia, Xianlin Liu, Jing Miao, Wei Xiong, Zilu Chen

**Affiliations:** a College of Chemistry and Chemical Engineering, Guangxi Normal University, Yucai Road 15, Guilin 541004, People’s Republic of China

## Abstract

The title compound, C_10_H_10_N_2_^2+^·C_10_H_8_O_4_^2−^, has inversion centres located at the geometric centres of the 1,4-phenyl­enediacetate anion and 4,4′-bipyridinium cation. The anions and cations are connected by N—H⋯O hydrogen bonds, forming one-dimensional supra­molecular chains, which inter­act with each other *via* π–π inter­actions [centroid–centroid distance = 3.938 (2) Å], building a two-dimensional supra­molecular sheet.

## Related literature

For related complexes of 1,4-phenyl­enediacetic acid, see: Braverman & LaDuca (2007 ▶); Soares-Santos *et al.* (2008 ▶); Liu *et al.* (2009 ▶); Chen *et al.* (2006*a* ▶,*b* ▶).
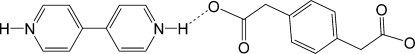


## Experimental

### 

#### Crystal data

C_10_H_10_N_2_^2+^·C_10_H_8_O_4_^2−^
*M* *_r_* = 350.36
Triclinic, 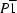

*a* = 4.579 (3) Å
*b* = 6.950 (5) Å
*c* = 13.859 (10) Å
α = 99.618 (9)°
β = 93.672 (9)°
γ = 97.373 (8)°
*V* = 429.6 (5) Å^3^
*Z* = 1
Mo *K*α radiation
μ = 0.10 mm^−1^
*T* = 296 K
0.25 × 0.20 × 0.18 mm

#### Data collection

Bruker SMART CCD area detector diffractometer
Absorption correction: multi-scan (*SADABS*; Bruker, 1998 ▶) *T*_min_ = 0.977, *T*_max_ = 0.983
2241 measured reflections
1499 independent reflections
1052 reflections with *I* > 2σ(*I*)
*R*_int_ = 0.014

#### Refinement

*R*[*F* ^2^ > 2σ(*F* ^2^)] = 0.058
*wR*(*F* ^2^) = 0.193
*S* = 1.07
1499 reflections
118 parameters
H-atom parameters constrained
Δρ_max_ = 0.41 e Å^−3^
Δρ_min_ = −0.25 e Å^−3^

### 

Data collection: *SMART* (Bruker, 2004 ▶); cell refinement: *SAINT* (Bruker, 2004 ▶); data reduction: *SAINT*; program(s) used to solve structure: *SHELXS97* (Sheldrick, 2008 ▶); program(s) used to refine structure: *SHELXL97* (Sheldrick, 2008 ▶); molecular graphics: *SHELXTL* (Sheldrick, 2008 ▶); software used to prepare material for publication: *SHELXTL*.

## Supplementary Material

Crystal structure: contains datablocks global, I. DOI: 10.1107/S1600536809036836/jh2103sup1.cif

Structure factors: contains datablocks I. DOI: 10.1107/S1600536809036836/jh2103Isup2.hkl

Additional supplementary materials:  crystallographic information; 3D view; checkCIF report

## Figures and Tables

**Table 1:** Hydrogen-bond geometry (Å, °)

*D*—H⋯*A*	*D*—H	H⋯*A*	*D*⋯*A*	*D*—H⋯*A*
N1—H1⋯O2	0.86	1.75	2.613 (3)	176

**Fig. 1.**
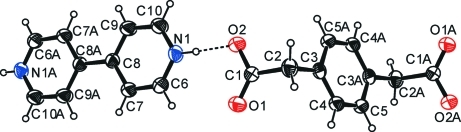
The molecular structure of the title compound with the atom-numbering scheme and 30% displacement ellipsoids.

**Fig. 2.**
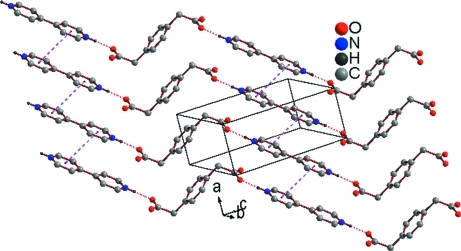
A view of the two-dimensional supramolecular sheet assembled by hydrogen bonds and π···π stacking interactions.

## supplementary crystallographic information

### Comment

The flexible ligand of 1,4-phenylenediacetate is drawing much interest in constructing metal–organic framework or supramolecular molecules due to its flexibility and its multifunctional groups of carboxylato group and phenyl ring (Braverman & LaDuca 2007; Soares-Santos *et al.*, 2008; Liu *et al.*, 2009; Chen *et al.*, 2006*a*,*b*). We thus report here a supramolecular structrure formed by 4,4'-bipyridine and 1,4-phenylenediacetic acid. The title compound 4,4'-bipyridinium 1,4-phenylenediacetate (Fig. 1), [C_10_H_10_N_2_][C_10_H_8_O_4_], has inversion centres located on the geometric centres of the 1,4-phenylenediacetate anion and 4,4'-bipyridinium cation. Each 4,4'-bipyridinium cation connects two 1,4-phenylenediacetate anions *via* N—H···O hydrogen bonds (Table 1), and *vice versa*. This leads to the formation of one dimensional supramolecular chains as shown in Fig. 2. The neighboring pyridyl rings from the adjacent one chains parallel to each other with perpendicular distance of 3.5654 (4) Å, a centre-to-centre distance of 3.938 (2) Å and an off-set angle of 25.135 (7)°. These information suggest the existence of significant π···π stacking interaction between the two pyridyl rings, which results in the construction of two dimensional supramolecular sheets (Fig. 2) from the one dimensional chains.

### Experimental

A mixture of 1,4-phenylenediacetic acid (0.0584 g, 0.3 mmol), 4,4'-bipyridine (0.0312 g, 0.2 mmol), Mn(CH_3_COO)_2_.4H_2_O (0.0735 g, 0.3 mmol) and ethanol (2 ml) was sealed in a 23 ml Teflon-lined autoclave, heated at 140 °C for 4 d and cooled over a period of 48 h. Colorless crystals of the title compound were collected in a yield of 60% (0.0802 g). Found: C, 68.26; H, 5.40; N, 7.28. C_20_H_18_N_2_O_4_ requires C, 68.56; H, 5.18; N, 7.52%.

### Refinement

H atoms on the carbon and nitrogen atoms were placed at calculated positions (C–H = 0.93 Å and N–H = 0.86 Å) and were included in the refinement in the riding model approximation, with *U*_iso_(H) = 1.2*U*_eq_(C, N).

### Crystal data

**Table:**

C_10_H_10_N_2_^2+^·C_10_H_8_O_4_^2^^−^	*Z* = 1
*M**_r_* = 350.36	*F*(000) = 184
Triclinic, *P*1	*D*_x_ = 1.354 Mg m^−^^3^
Hall symbol: -P 1	Mo *K*α radiation, λ = 0.71073 Å
*a* = 4.579 (3) Å	Cell parameters from 736 reflections
*b* = 6.950 (5) Å	θ = 3.0–23.8°
*c* = 13.859 (10) Å	µ = 0.10 mm^−^^1^
α = 99.618 (9)°	*T* = 296 K
β = 93.672 (9)°	Block, colourless
γ = 97.373 (8)°	0.25 × 0.20 × 0.18 mm
*V* = 429.6 (5) Å^3^	

### Data collection

**Table:**

Bruker SMART CCD area detector diffractometer	1499 independent reflections
Radiation source: fine-focus sealed tube	1052 reflections with *I* > 2σ(*I*)
graphite	*R*_int_ = 0.014
φ and ω scans	θ_max_ = 25.0°, θ_min_ = 3.0°
Absorption correction: multi-scan (*SADABS*; Bruker, 1998)	*h* = −5→5
*T*_min_ = 0.977, *T*_max_ = 0.983	*k* = −8→7
2241 measured reflections	*l* = −10→16

### Refinement

**Table:**

Refinement on *F*^2^	Primary atom site location: structure-invariant direct methods
Least-squares matrix: full	Secondary atom site location: difference Fourier map
*R*[*F*^2^ > 2σ(*F*^2^)] = 0.058	Hydrogen site location: inferred from neighbouring sites
*wR*(*F*^2^) = 0.193	H-atom parameters constrained
*S* = 1.07	*w* = 1/[σ^2^(*F*_o_^2^) + (0.0979*P*)^2^ + 0.1429*P*] where *P* = (*F*_o_^2^ + 2*F*_c_^2^)/3
1499 reflections	(Δ/σ)_max_ < 0.001
118 parameters	Δρ_max_ = 0.41 e Å^−^^3^
0 restraints	Δρ_min_ = −0.25 e Å^−^^3^

### Special details

**Table:**

Geometry. All e.s.d.'s (except the e.s.d. in the dihedral angle between two l.s. planes) are estimated using the full covariance matrix. The cell e.s.d.'s are taken into account individually in the estimation of e.s.d.'s in distances, angles and torsion angles; correlations between e.s.d.'s in cell parameters are only used when they are defined by crystal symmetry. An approximate (isotropic) treatment of cell e.s.d.'s is used for estimating e.s.d.'s involving l.s. planes.
Refinement. Refinement of *F*^2^ against ALL reflections. The weighted *R*-factor *wR* and goodness of fit *S* are based on *F*^2^, conventional *R*-factors *R* are based on *F*, with *F* set to zero for negative *F*^2^. The threshold expression of *F*^2^ > σ(*F*^2^) is used only for calculating *R*-factors(gt) *etc*. and is not relevant to the choice of reflections for refinement. *R*-factors based on *F*^2^ are statistically about twice as large as those based on *F*, and *R*- factors based on ALL data will be even larger.

### Fractional atomic coordinates and isotropic or equivalent isotropic displacement parameters (Å^2^)

**Table:**

	*x*	*y*	*z*	*U*_iso_*/*U*_eq_	
O2	0.6746 (5)	0.5442 (3)	0.19005 (15)	0.0691 (7)	
O1	0.7402 (6)	0.3340 (3)	0.28869 (16)	0.0812 (8)	
N1	0.3813 (5)	0.7303 (4)	0.32281 (18)	0.0628 (7)	
H1	0.4734	0.6645	0.2794	0.075*	
C8	0.0807 (6)	0.9428 (4)	0.46281 (19)	0.0511 (7)	
C4	0.6539 (6)	−0.0571 (4)	0.0761 (2)	0.0568 (8)	
H4	0.7572	−0.0977	0.1273	0.068*	
C5	0.4446 (7)	−0.1901 (4)	0.0152 (2)	0.0583 (8)	
H5	0.4078	−0.3191	0.0262	0.070*	
C3	0.7125 (6)	0.1349 (4)	0.06220 (19)	0.0522 (7)	
C7	0.1869 (7)	0.7720 (4)	0.4784 (2)	0.0644 (8)	
H7	0.1595	0.7255	0.5367	0.077*	
C9	0.1311 (8)	1.0030 (5)	0.3742 (2)	0.0709 (9)	
H9	0.0632	1.1163	0.3597	0.085*	
C6	0.3343 (7)	0.6709 (4)	0.4066 (2)	0.0689 (9)	
H6	0.4032	0.5558	0.4179	0.083*	
C2	0.9333 (7)	0.2827 (5)	0.1320 (2)	0.0642 (8)	
H2A	1.0804	0.2150	0.1606	0.077*	
H2B	1.0338	0.3765	0.0963	0.077*	
C1	0.7762 (6)	0.3900 (4)	0.2123 (2)	0.0524 (7)	
C10	0.2832 (7)	0.8930 (5)	0.3077 (2)	0.0739 (10)	
H10	0.3182	0.9365	0.2490	0.089*	

### Atomic displacement parameters (Å^2^)

**Table:**

	*U*^11^	*U*^22^	*U*^33^	*U*^12^	*U*^13^	*U*^23^
O2	0.0924 (16)	0.0590 (13)	0.0641 (14)	0.0268 (11)	0.0119 (11)	0.0198 (10)
O1	0.119 (2)	0.0764 (15)	0.0619 (14)	0.0402 (14)	0.0238 (13)	0.0264 (12)
N1	0.0687 (16)	0.0644 (16)	0.0565 (15)	0.0193 (12)	0.0133 (12)	0.0033 (12)
C8	0.0531 (16)	0.0494 (15)	0.0509 (16)	0.0097 (12)	0.0035 (12)	0.0074 (12)
C4	0.0694 (18)	0.0581 (17)	0.0498 (16)	0.0235 (14)	0.0140 (14)	0.0151 (13)
C5	0.0774 (19)	0.0476 (16)	0.0568 (17)	0.0191 (14)	0.0214 (15)	0.0157 (13)
C3	0.0563 (16)	0.0547 (16)	0.0487 (15)	0.0168 (13)	0.0214 (12)	0.0049 (12)
C7	0.085 (2)	0.0583 (18)	0.0566 (18)	0.0252 (15)	0.0140 (15)	0.0156 (14)
C9	0.094 (2)	0.066 (2)	0.066 (2)	0.0364 (17)	0.0240 (17)	0.0240 (15)
C6	0.092 (2)	0.0569 (18)	0.065 (2)	0.0303 (17)	0.0141 (17)	0.0125 (15)
C2	0.0603 (17)	0.0675 (19)	0.0650 (19)	0.0138 (15)	0.0152 (14)	0.0048 (15)
C1	0.0589 (16)	0.0480 (15)	0.0503 (16)	0.0081 (13)	0.0052 (13)	0.0081 (12)
C10	0.095 (2)	0.076 (2)	0.063 (2)	0.0354 (19)	0.0246 (18)	0.0229 (16)

### Geometric parameters (Å, °)

**Table:**

O2—C1	1.296 (3)	C5—H5	0.9300
O1—C1	1.202 (3)	C3—C5^ii^	1.388 (4)
N1—C10	1.312 (4)	C3—C2	1.513 (4)
N1—C6	1.317 (4)	C7—C6	1.383 (4)
N1—H1	0.8600	C7—H7	0.9300
C8—C7	1.383 (4)	C9—C10	1.379 (4)
C8—C9	1.386 (4)	C9—H9	0.9300
C8—C8^i^	1.488 (5)	C6—H6	0.9300
C4—C3	1.375 (4)	C2—C1	1.508 (4)
C4—C5	1.375 (4)	C2—H2A	0.9700
C4—H4	0.9300	C2—H2B	0.9700
C5—C3^ii^	1.388 (4)	C10—H10	0.9300
			
C10—N1—C6	117.9 (3)	C10—C9—C8	119.3 (3)
C10—N1—H1	121.0	C10—C9—H9	120.4
C6—N1—H1	121.0	C8—C9—H9	120.4
C7—C8—C9	116.8 (3)	N1—C6—C7	122.9 (3)
C7—C8—C8^i^	122.0 (3)	N1—C6—H6	118.5
C9—C8—C8^i^	121.3 (3)	C7—C6—H6	118.5
C3—C4—C5	120.9 (3)	C1—C2—C3	109.8 (2)
C3—C4—H4	119.5	C1—C2—H2A	109.7
C5—C4—H4	119.5	C3—C2—H2A	109.7
C4—C5—C3^ii^	121.1 (3)	C1—C2—H2B	109.7
C4—C5—H5	119.5	C3—C2—H2B	109.7
C3^ii^—C5—H5	119.5	H2A—C2—H2B	108.2
C4—C3—C5^ii^	118.0 (3)	O1—C1—O2	123.0 (3)
C4—C3—C2	120.7 (3)	O1—C1—C2	123.0 (3)
C5^ii^—C3—C2	121.3 (3)	O2—C1—C2	113.9 (3)
C8—C7—C6	119.6 (3)	N1—C10—C9	123.5 (3)
C8—C7—H7	120.2	N1—C10—H10	118.3
C6—C7—H7	120.2	C9—C10—H10	118.3
			
C3—C4—C5—C3^ii^	0.3 (4)	C8—C7—C6—N1	0.4 (5)
C5—C4—C3—C5^ii^	−0.3 (4)	C4—C3—C2—C1	−92.6 (3)
C5—C4—C3—C2	177.3 (2)	C5^ii^—C3—C2—C1	85.0 (3)
C9—C8—C7—C6	−0.2 (5)	C3—C2—C1—O1	90.7 (4)
C8^i^—C8—C7—C6	179.7 (3)	C3—C2—C1—O2	−87.0 (3)
C7—C8—C9—C10	−0.5 (5)	C6—N1—C10—C9	−0.8 (5)
C8^i^—C8—C9—C10	179.6 (3)	C8—C9—C10—N1	1.0 (6)
C10—N1—C6—C7	0.1 (5)		

Symmetry codes: (i) −*x*, −*y*+2, −*z*+1; (ii) −*x*+1, −*y*, −*z*.

### Hydrogen-bond geometry (Å, °)

**Table:**

*D*—H···*A*	*D*—H	H···*A*	*D*···*A*	*D*—H···*A*
N1—H1···O2	0.86	1.75	2.613 (3)	176
